# The Effectiveness of Electroconvulsive Therapy on Catatonia in a Case of Anti-N-Methyl-D-Aspartate (Anti-NMDA) Receptor Encephalitis

**DOI:** 10.7759/cureus.15706

**Published:** 2021-06-17

**Authors:** Kehinde T Olaleye, Adeolu O Oladunjoye, David Otuada, Gibson O Anugwom, Tajudeen O Basiru, Jennifer E Udeogu, Taiwo Opaleye-Enakhimion, Eduardo D Espiridion

**Affiliations:** 1 Neurology, Tampa General Hospital, Tampa, USA; 2 Psychiatry, Reading Hospital Tower Health, West Reading, USA; 3 Medical Critical Care, Boston Children's Hospital, Boston, USA; 4 Psychiatry and Behavioral Sciences, West Oaks Behavioral Hospital, Houston, USA; 5 Psychiatry and Behavioral Sciences, Houston Behavioral Healthcare Hospital, Houston, USA; 6 Developmental Behavioral Pediatrics, Dell Children's Medical Center, Austin, USA; 7 Public Health, Georgia Department of Public Health, Savannah, USA; 8 Public Health, Greater Philadelphia Health Action, Philadelphia, USA; 9 Psychiatry, Drexel University College of Medicine, Philadelphia, USA; 10 Psychiatry, West Virginia School of Osteopathic Medicine, Lewisburg, USA; 11 Psychiatry, West Virginia University School of Medicine, Martinsburg, USA; 12 Psychiatry, Philadelphia College of Osteopathic Medicine, Philadelphia, USA

**Keywords:** electro-convulsive therapy, bush francis catatonia scale, catatonia, anti nmda receptor encephalitis, encephalitis

## Abstract

Catatonia is a symptom seen in a variety of neuropsychiatric conditions, including anti-N-Methyl D-aspartate receptor (NMDAR) encephalitis. When associated with anti-NMDAR encephalitis, catatonia is resistant to standard therapy. However, electroconvulsive therapy (ECT) has shown promising success in management. This case report presents a 25-year-old African American female who presented to the emergency room with nervousness, sweating, insomnia, and visual and auditory hallucinations. She was treated symptomatically for anxiety but returned to the hospital after she continued to experience worsening symptoms. Her anxiety worsened, and she became more agitated, warranting an extensive workup, including magnetic resonance imaging (MRI) and electroencephalogram (EEG), which showed normal findings. She also had an anti-NMDA receptor antibodies titer done, which showed a positive titer result. She was treated with intravenous steroids, intravenous immunoglobulin G (IgG), plasma exchange, and rituximab, which did not improve her symptoms, and she was discharged home after a prolonged hospital stay. On follow-up visits, she reported worsening confusion, aggression, and suicidal behaviors. The patient was readmitted, during which she experienced catatonia and psychiatric symptoms, and her anti-NMDAR titer had increased to 1:1280. Further treatments with intravenous steroids, intravenous IgG, plasma exchange, and rituximab, including haloperidol and clonazepam, failed to improve her condition. However, her condition improved remarkably following treatment with 12 rounds of ECT. No randomized control trial has been done to demonstrate the effectiveness of ECT in the treatment of anti-NMDAR encephalitis despite various reports of the effectiveness of this treatment modality. This case report adds to the growing clinical evidence in support of the use of ECT in anti-NMDAR encephalitis patients with catatonia. ECT can be incorporated as standard protocol in the treatment of catatonia and associated psychiatric symptoms when managing a patient with anti-NMDAR encephalitis associated with catatonic features.

## Introduction

Anti-N-methyl-D-aspartate receptor (NMDAR) encephalitis is a rare autoimmune encephalitis that was first described by Josep Dalmau and his colleague in 2007 [1]. It is suggested to be the most common autoimmune cause of encephalitis after demyelinating encephalitis [2]. Although anti-NMDAR encephalitis can occur at any age and in both sexes, about 80% of cases are females, mostly 18 years and younger [3]. In many published cases, patients have underlying tumors, with ovarian teratomas being the most implicated [3-4]. Frequently, symptoms of anti-NMDAR encephalitis manifest with acute neuropsychiatric presentations such as mania, visual and auditory hallucinations, personality change, cognitive decline, catatonia, and new-onset behavioral problems [3,5-6]. 

The management of anti-NMDAR encephalitis is challenging, as its neuropsychiatric manifestations often mask the underlying etiology. This is especially challenging for psychiatrists who are usually the first to assess approximately 77% of these patients [3,7] and can be treated as a case of schizophrenia because of the similar presentation of psychotic symptoms in NMDAR encephalitis [4,8]. The diagnosis is confirmed by identifying anti-NMDAR antibodies in cerebrospinal fluid (CSF) [1,5]. It has been suggested that early treatment is essential, especially in children [9-10], which is why prompt diagnosis is paramount. Presently, the mortality rate is as high as 7%, and the chances of complete recovery decrease with disease progression [3]; therefore, it is important that the treatment options for NMDAR encephalitis are effective [5]. The current treatment options available for the underlying etiology include plasmapheresis and immunomodulatory agents. Based on current evidence, immunotherapy alone does not adequately treat this condition. Available literature suggests that electroconvulsive therapy (ECT) improves the associated symptoms of stupor, catatonia, mutism, delusions, and psychosis [4,8,11]. Very few studies describe the consistent use of ECT for anti-NMDA encephalitis with catatonia and its associated psychiatric symptoms.

In this report, we present a case of a 25-year-old female with severe psychiatric symptoms, including psychotic symptoms, aggressive symptoms, and catatonia, despite treatment with immunotherapy, plasma exchange, rituximab, and intravenous (IV) steroids. Currently, there is limited information in the literature regarding the consistent use of ECT in the management of the acute, debilitating neuropsychiatric symptoms of anti-NMDAR encephalitis with catatonia.

## Case presentation

History of present illness

A 25-year-old African American female with no known past medical history or psychiatric history was brought to the hospital by her mother due to changes in her behavior (nervousness, sweating, lack of sleep) and palpitation. The patient experienced auditory and visual hallucinations (yelled at people she alone could see to keep them away from her). The patient's mother stated that she thought the patient was suicidal when she heard her saying, "I am going to die," prompting her to call 911. However, there was no evidence of suicidality at this time. The patient's mother reported the patient's occasional use of cannabis and alcohol. At the emergency room (ER), a diagnosis of anxiety was made, and she was treated and discharged home on hydroxyzine 25 mg every six hours. However, her symptoms persisted and even worsened despite compliance with medications. She spent the next two days in her room, unable to sleep, anxious, and repeating the exact words, "you are going to be okay." Her anxiety worsened and became more agitated warranting readmission and an extensive workup that included anti-NMDA receptor antibodies showing an NMDA titer of 1:320 in her CSF and confirming anti-NMDA receptor encephalitis. IV steroids, plasma exchange, and rituximab were then administered during this patient's long hospital stay. She completed numerous lab tests, including brain magnetic resonance imaging (MRI) and electroencephalogram (EEG), which were negative. She was then discharged home for later follow-up visits.

After two days, she returned to the hospital with a report of worsening combativeness, confusion, and aggression at home. She exhibited suicidal behaviors, including possessing a razor blade to hurt herself and overdosing on a handful of unspecified medications. NMDA titers were repeated and showed increased levels of 1:1280. IV immunoglobulins (IVIG) and intravenous methylprednisolone and rituximab were repeated. She was later transferred to a higher level care center due to the concern of no improvement in her aggression and combativeness with new features of catatonia, including mutism, sitting abnormally still, staring, catalepsy, waxy flexibility, occasional impulsivity, and occasional aggression. At this center, she received lorazepam for 10 days with no significant improvement in her symptoms and was then commenced on emergency ECT treatment. Catatonia due to another medical condition was considered during the patient's treatment course. The differential diagnoses considered during her treatment were hepatitis B virus, primary central nervous system vasculitis, neuroleptic malignant syndrome, and hypersensitivity to first-generation antipsychotics such as haloperidol. The possibility of neuropsychiatric systemic lupus erythematosus (SLE) was considered in this patient given the sociodemographic and symptomatic profile but the physicians who managed this patient did not consider her as having SLE.

Examination

During the assessment, the patient was alert and mute but followed simple commands and nodded yes/no in response to questions. Pupils were equal and reactive to light; she blinked to threat in all visual fields. Intact extraocular movements without nystagmus or ptosis were observed. Her facial sensations were intact and symmetric to light touch on the V1-V3 distribution. Her face was symmetric with a smile and tight eye closure. Uvula and palate rose midline. Shoulder shrug/head turns were symmetric with 5/5 strength. The tongue was midline with no protrusion. There was an increased tone in all her four extremities but no abnormal movements. Strength in all extremities was ⅗, and reflexes of 2+ were present symmetrically in her biceps, brachioradialis, patellar, and Achilles. However, she exhibited catatonic features, which lasted from minutes to hours. Her Bush-Francis Catatonia Rating Scale (BFCRS) was 13 (1 for mobility, 3 for mutism, 2 for staring, 1 for posturing, 3 for waxy flexibility, 1 for impulsivity, 1 for combativeness).

Mental status examination revealed a young lady, well-groomed, with casual wear. She was cooperative and calm. She exhibited minimal psychomotor retardation with a lack of movement. Her speech was slow, with a latency of about 5 seconds. Her affect was flat and mood dysphoric. She was awake, alert, and oriented to time, place, person, and event. Her thought process was linear and concrete. Her judgment and insight were fair.

Investigation

The following investigations were completed during her latest hospital stay. Complete metabolic panel showed; sodium 137 meq/l (normal range: 135 - 148 meq/l), potassium 4.4 meq/l (normal range: 3.5 - 5.3 meq/l), chloride 102 meq/l (normal range: 98 - 107 meq/l), Co2 28 meq/l (normal range: 22 - 29 meq/l), blood urea nitrogen (BUN) 16 mg/dl (normal range: 6 - 20 mg/dl), serum glucose 100 mg/dl (normal range: 70 - 110 mg/dl), blood creatinine 0.9 mg/dl (normal range: 0.57 - 1.11 mg/dl), calcium 9.1 mg/dl (normal range: 8.5 - 10.5 mg/dl), anion gap 7 meq/l (normal range: 5 - 13 meq/l), BUN/creatinine ratio 18 (normal range: 10 - 20). Complete blood count (CBC) showed white blood cell (WBC) 17.55 103/ul (normal range: 4.6 - 10.2 10^3^/ul), RBC 4.77 10^6^/ul (normal range: 4.04 - 5.48 10^6^/ul), hemoglobin 11.9 g/dl (normal range: 12.2 - 16.2 g/dl), hematocrit 37.6% (normal range: 37.7 - 47.9%), mean corpuscular volume (MCV) 78.8 fl (normal range: 80 - 97 fl), mean corpuscular hemoglobin (MCH) 24.9 pg (normal range: 27.0 - 31.2 pg), mean corpuscular hemoglobin concentration (MCHC) 31.6 g/dl (normal range: 31.8 - 35.4 g/dl), platelet count 508 X 10^3^/ul (normal range: 142.0 - 424.0 X 10^3^/ul), mean platelet volume (MPV) 10.2 fl (normal range: 9.4 - 12.4 fl), red cell distribution width (RDW) 17.3% (normal range: 11.6 - 14.6%). Thyriod stimulating hormone (TSH) was 1.03 mIU/L (0.5 to 5.0 mIU/L). The hepatitis B viral panel showed a negative hepatitis B surface antigen and positive hepatitis B core antibody. Malignancy work-up, including transvarginal ultrasound and pelvic MRI, was negative for any malignancy ruling out ovarian teratoma. There were no suspicious adnexal masses.

**Figure 1:**
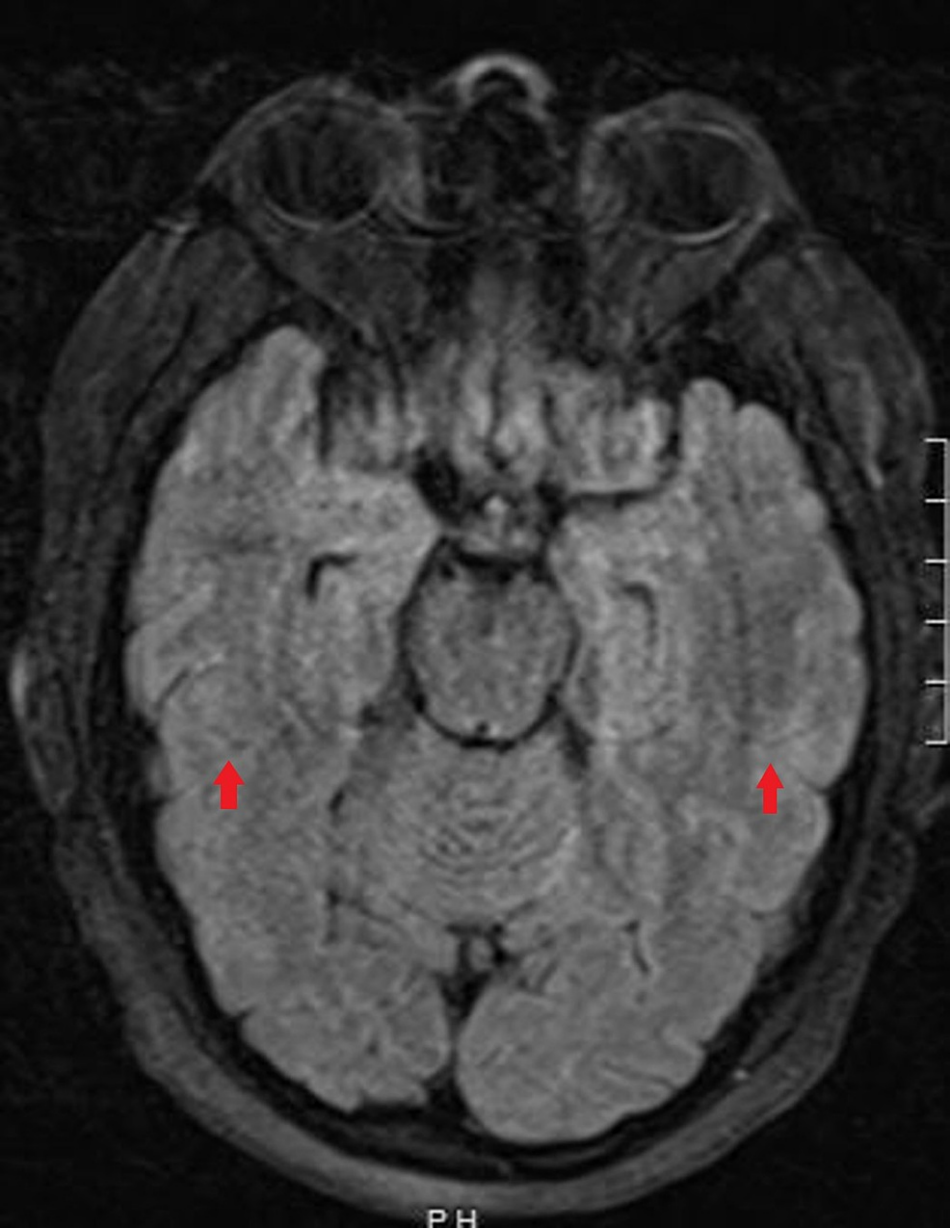
Magnetic resonance imaging (MRI) of the brain Normal brain MRI with no abnormal leptomeningeal or intraparenchymal enhancement (red arrows). No hemorrhage, mass, or acute infarct seen.

Anti-NMDAR antibodies were positive at the most recent visit with a serum NMDA titer increase to 1:1280 from the previous 1:320 at the initial diagnosis of NMDAR encephalitis. A brain MRI showed no mass lesion, hemorrhage, or acute infarct. There was no leptomeningeal or intraparenchymal enhancement (Figure 1). The ventricular system, cisterns, and sulci were of normal size, shape, and contour. EEG showed normal findings. Other imaging investigations done were CT chest, abdomen, and pelvis, all of which showed no primary malignancy or metastatic lesion.

Treatment 

At her initial visit, after the diagnosis of anti-NMDAR encephalitis, she received intravenous immunoglobulins (IVIG) and intravenous methylprednisolone. However, when the NMDA titer was found to be elevated, the decision was made to repeat IVIG, intravenous methylprednisolone, and add rituximab. She received IVIG 2 g/kg over five days, was continued on rituximab and IVIG 1 g. Haloperidol 5 mg three times daily, memantine 5 mg daily, clonazepam 1 mg three times daily, and gabapentin. During her hospitalization, she had extensive long-term electroencephalographic monitoring that did not disclose any seizure activity.

Further immunotherapies were considered. The patient received a third dose of rituximab and was started on prednisone 60 mg daily. Due to concerns for catatonia, lorazepam 2 mg every six hours was commenced and titrated to a high dose. Because she had only a minimal response, amantadine 100 mg twice a day was added. Nonetheless, these treatments proved to be of limited benefit, as the patient remained symptomatic while on admission for about 10 days. An emergent ECT was performed, and she subsequently had 12 ECT sessions, after which her BFCRS score decreased to 0 with significant improvement in her catatonia, cognitive function, and overall mental status. The patient was started on a prolonged prednisone taper and was later discharged on 40 mg four times daily, with instructions on tapering it down. She was recommended for follow-up with psychiatry and neurology.

## Discussion

Psychiatric symptoms are the most common presentation in patients with anti-NMDAR encephalitis, with an incidence of about 65%-80% [1,12]. Most patients with anti-NMDAR encephalitis, as seen in this patient, will present with catatonia during their illness [1]. Benzodiazepine has been identified as a safe and effective treatment for catatonia, and a study by Petrides et al. proposed that once benzodiazepine is ineffective for catatonia, ECT should be commenced immediately in these patients to improve clinical outcomes [13]. They propose that the treatment algorithm should use benzodiazepine and/or ECT in addition to immunotherapy to target antibody development [13].

This case adds to available literature in which ECT was found to be very effective in the treatment of catatonia in patients with anti-NMDAR encephalitis [13-14]. A recent systematic review in which young adult patients with anti-NMDAR encephalitis complicated by severe psychiatric symptoms (primarily catatonia) significantly improved with ECT, with complete (60%) or partial (30%) recovery of the illness [14]. These cases, including ours, represent a breakthrough in treating a newly described autoimmune encephalitis diagnosis that has been challenging to manage for years. Some studies propose that benzodiazepine or ECT should be used to augment first-line treatment like immunotherapy when treating catatonia, a severe psychiatric feature of anti-NMDAR encephalitis [4,6]. Unlike our patient, who received several rounds of first-line therapy, immunotherapy, and plasmapheresis, and then second-line therapy, rituximab, with no significant improvement, some studies have found ECT treatment complementing treatment with plasmapheresis and/or immunotherapy to be beneficial. Our patient's catatonic symptoms only resolved after ECT was commenced, leading to the complete resolution of symptoms. 

Nevertheless, ECT is still essential to prevent worsening clinical conditions such as catatonia, dysautonomia, and psychotic features in anti-NMDAR encephalitis patients. Catatonia is a severe and life-threatening neuropsychiatric syndrome associated with multiple medical disorders, including anti-NMDAR encephalitis. ECT is thought to help GABAergic enhancement in the CNS, leading to anti-catatonic action [15]. A study proposed that ECT may be responsible for the regeneration of autoantibody-damaged NMDAR in the hippocampus by improving its glutamate subunit binding [16]. It is reported that ECT improves patients' recovery and leads to shorter hospital lengths of stay [4]. Our patient improved after 12 rounds of ECT treatment, with full recovery of her catatonic symptoms.

One of the challenges in managing anti-NMDAR encephalitis is the lack of well-established diagnostic criteria and the long duration, which may take weeks to months before identifying NMDAR antibodies in CSF and blood [17]. When the antibodies are identified, the severity of anti-NMDAR encephalitis is closely associated with titer levels. It has been reported that patients who improve clinically have a concomitant decrease in titer levels and vice versa [1]. This finding is consistent with what was found in our patient whose clinical condition increased in severity as her anti-NMDAR antibody titer increased from 1:320 at initial diagnosis of anti-NMDAR encephalitis to 1:1280 before the commencement of ECT. Further research needs to be done to assess how best to monitor patients' improvement even though titers seem to correlate with the clinical progression of the illness [18]. Hence, a high index of suspicion and prompt treatment are needed to avoid misdiagnoses or delays in treatment, which may further worsen the clinical condition.

Due to the rarity of the disease, there are no clear-cut outlines of treatments, but as seen in our patient and other reported cases of anti- NMDAR encephalitis, ECT is very effective. A study reported that 26 of 30 cases of NMDAR encephalitis managed with ECT showed no safety concerns [14]. The four cases that had their ECT prematurely stopped made full recovery with immunotherapy. A transient cognitive side effect is the most concerning risk [19]. However, severe catatonia treated with ECT shows improvement in cognition as their catatonia decreases in response to ECT treatment [17,20].

In our case, there was no observed improvement in the patient's catatonia and other psychiatric symptoms with the initial administration of benzodiazepines, immunotherapies, and antipsychotic therapy. However, remarkable improvement in the catatonia and other psychiatric symptoms was seen after initiating ECT. Studies have also shown that incorporating ECT in the management of catatonia associated with anti-NMDAR encephalitis improves patient recovery, shortening recovery time, reducing patients' cost, and shortening hospital stay. As seen in our case report, ECT therapy represents a way to help patients return to normalcy. ECT is the most effective treatment for catatonia regardless of the etiology and, in particular, due to medical illness as is in this case.

## Conclusions

The successful remission of catatonia associated with anti-NMDAR encephalitis using ECT treatment in our patient adds to the growing list of successful treatments reported in previous studies. ECT should be incorporated as standard protocol in the management of catatonia associated with anti-NMDAR encephalitis used in treating catatonia and associated psychiatric symptoms seen in these patients. Early treatment can be associated with better clinical outcomes.
